# Kawasaki Disease Following Severe Sunburn Injury

**DOI:** 10.3389/fped.2020.00006

**Published:** 2020-01-28

**Authors:** Seigo Okada, Shintaro Hashimoto, Akiko Miyake, Yusuke Okada, Reiji Hirano, Shinnosuke Fukunaga, Yuichi Ishikawa

**Affiliations:** ^1^Department of Pediatrics, Saiseikai Shimonoseki General Hospital, Yamaguchi, Japan; ^2^Department of Pediatrics, Yamaguchi University Graduate School of Medicine, Yamaguchi, Japan

**Keywords:** damage-associated molecular patterns, innate immunity, Kawasaki disease, pathogen-associated molecular patterns, scaled injury

## Abstract

**Background:** Although an etiology of Kawasaki disease (KD) is unknown, an aberrant innate immune system in predisposed individuals has been proposed to play a key role in the development of KD vasculitis. Various etiological pathogens have been proposed as the trigger of KD and a scaled injury preceding symptom onset has been reported as one of them. Here, we report a 17-month-old Japanese female who was hospitalized due to high fever lasting for 4 days with infection ruled out as a cause. On admission, she displayed severe sunburn all over her body following a prolonged period of outdoor play 5 days ago. On the 5 day of illness, she developed complete KD. Serum levels of high mobility group box 1, a representative for damage-associated molecular patterns (DAMPs), were elevated during acute phase and continued to decrease during subacute phase. This unique course suggested the inflammatory process of KD involving innate immunity via DAMPs.

## Introduction

Kawasaki disease (KD) is an acute, febrile and systemic vasculitis that primarily occurs in infants and young children (1). Although its etiology is unknown, an aberrant innate immune system in predisposed individuals has been proposed to play a key role in the development of KD vasculitis (2, 3). Several studies suggest a possibility that KD-specific innate immune pathogen-associated molecular patterns (PAMPs)/microbe-associated molecular patterns (MAMPs) induce vascular inflammation, leading to the development of KD (2, 3). PAMPs/MAMPs also induce damage-associated molecular patterns (DAMPs) production, such as S100 proteins and high mobility group box 1 (HMGB1), by host cells (3). Previous studies reported that DAMPs were elevated in the sera of patients with KD during the acute phase (1, 4–8).

Various etiological pathogens have been proposed as the trigger of KD, but none have been decisively established (1). In previous reports, a scaled injury preceding symptom onset could potentially trigger KD (9–11). The thermal injury and the onset of KD could be explained by the entry of an infectious agent(s), toxin, or superantigen through a compromised skin barrier (1, 3, 9–11). DAMPs released after burn injury play a critical role in the activation of the innate immunity (12–14). Hence, released DAMPs due to burn injury might also be involved in inflammatory vasculitis and the clinical manifestations of KD (1, 3, 9, 10).

Here, we describe the case of a 17-month-old female who developed complete KD following scaled injury due to severe sunburn. Although the patient presented a severe ill condition on admission, she achieved complete defervescence along with the improvement of clinical manifestations after a single course of intravenous immunoglobulin (IVIG).

## Case Presentation

**Figure 1:**
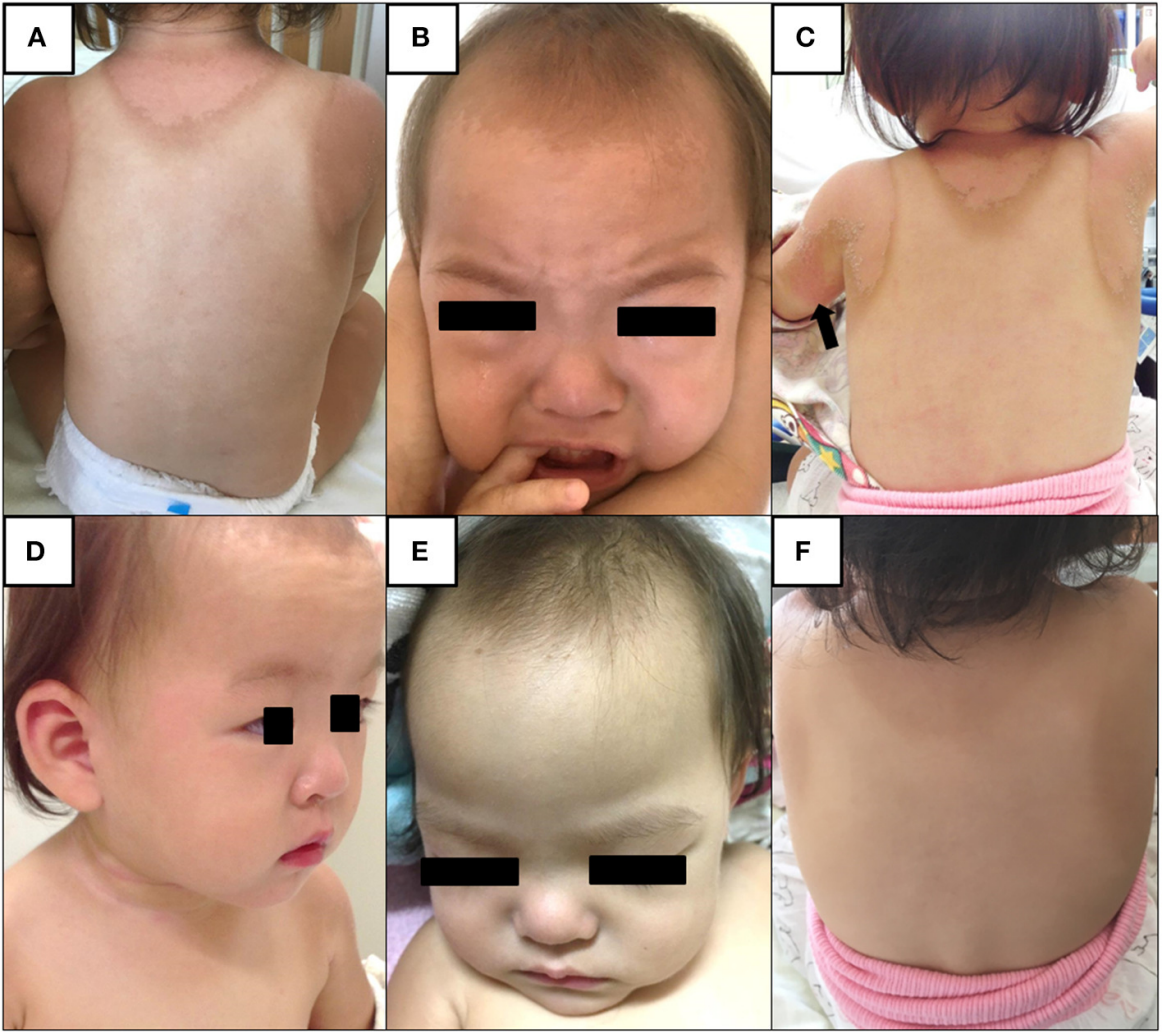
**(A,B)** On admission. Brunet sunburn marks along lines of clothes are left on the skin of body trunk and face due to severe sunburn injury. The patient's skins are peeling. **(C,D)** On the next day after admission (5th day of illness). The patient shows erythema of the trunk of the body, redness at the Bacille Calmette-Guérin inoculation site (*arrow*), conjunctival hyperemia, redness of the lips, strawberry tongue, and cervical lymphadenopathy. **(E,F)** On the day of discharge (15th day of illness). Brunet sunburn marks and distinctive symptoms of Kawasaki disease have disappeared.

**Figure 2:**
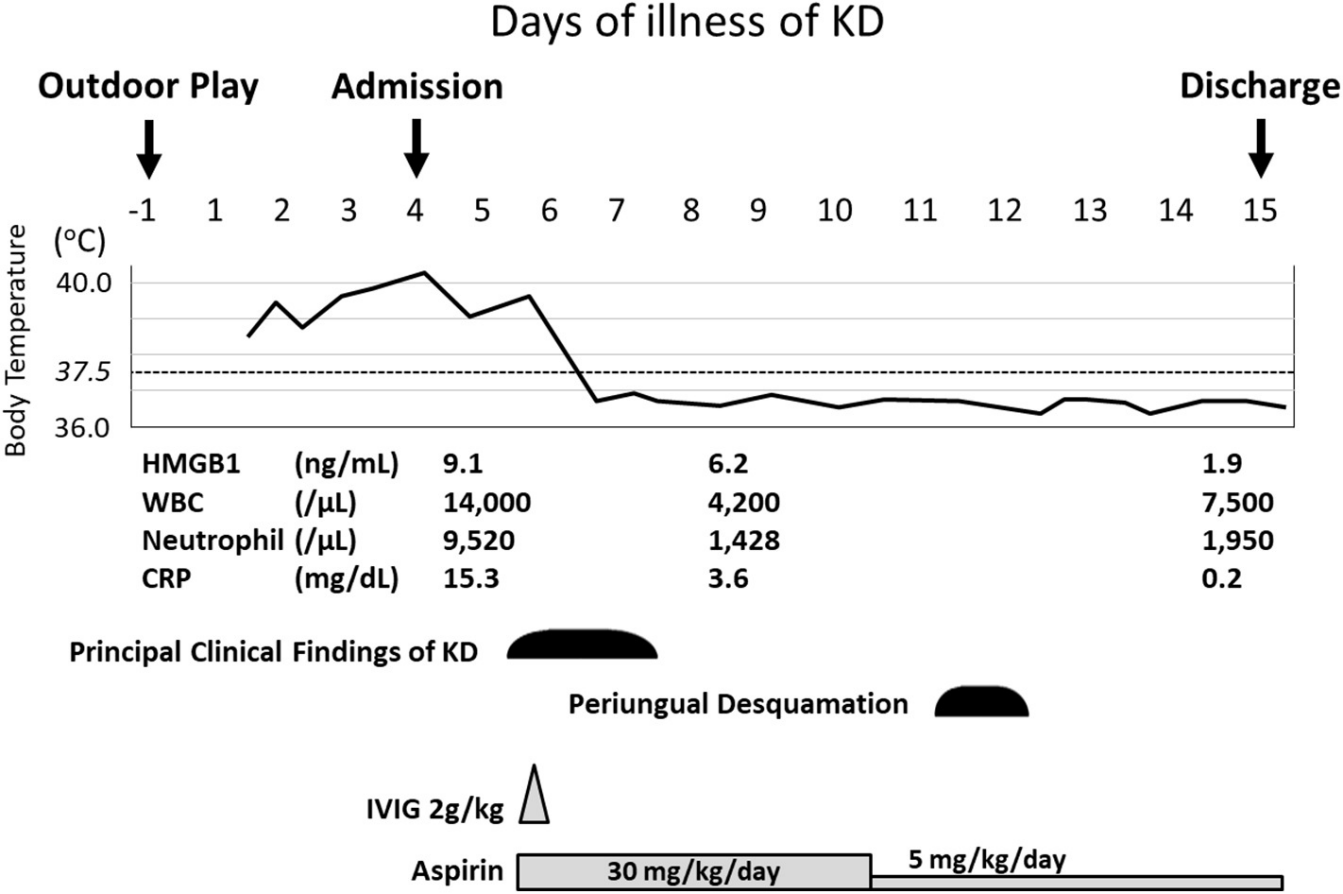
Clinical course of the present patient. The values of HMGB1 were determined on 4th day (before IVIG), 8th day (after IVIG), and 14th day (convalescent phase). CRP, C-reactive protein; HMGB1, high mobility group box 1; IVIG, intravenous immunoglobulin; KD, Kawasaki disease; WBC, white blood cell.

A 17-month-old Japanese female was hospitalized due to high fever lasting for 4 days in August 2018. The present patient was born to non-consanguineous healthy parents as an extremely premature infant at 26 weeks of gestation with a birth weight of 956 g. She was well-developed after discharge from the neonatal intensive care unit. On admission, her height and weight were 75.0 cm (0.2 standard deviation [SD] for corrected age) and 9.3 kg (0.3 SD for corrected age), respectively. She was not on any medications. She had no contact with potential sources of infection in the 2 weeks prior to her admission. Her family members had been all well during the period. At the initial visit, she showed high fever of 40.4°C with infection ruled out as a cause. She displayed severe sunburn on large area of her body; first- and second-degree thermal skin injuries accounted for 32 and 29% of her body, respectively (Figures 1A,B). Brunet sunburn marks along lines of clothes were left on the skin of body trunk and face. The patient's skins where small bullae had been existed were peeling. Her mother mentioned that she had the sunburn following a 3 h outdoor play on the bathing beach without sunscreen 5 days ago. Maximum ultraviolet index on the day was 9 (very high). She did not receive any treatment for the sunburn. Blood examination showed a white blood cell (WBC) count of 14.0 × 10^9^/L, with 68.0% segmented neutrophils and C-reactive protein (CRP) concentration of 15.3 mg/dL. Urinalysis and abdominal ultrasonography results were normal and rapid diagnostic test for adenovirus was negative. She was hospitalized and received intravenous cefotaxime, but symptoms did not improve. On the next day of hospitalization (5th day of illness), conjunctival hyperemia, erythema of the trunk of the body, redness of the lips, strawberry tongue, cervical lymphadenopathy, and redness at the Bacille Calmette-Guérin inoculation site emerged (Figures 1C,D). Fulfillment of the diagnostic criteria of KD prompted us to start 2 g/kg of IVIG and oral aspirin. Single dose IVIG led to a prompt response with defervescence. She was discharged from the hospital on the 15th day of illness without coronary artery lesions (CALs) (Figures 1E,F). Nose, throat, blood, urine, and stool cultures identified no causative bacteria. After her discharge, we measured the HMGB1, as a representative for DAMPs, in the conserved sera using an ELISA kit (HMGB1 ELISA kit II; Shino-test Corporation, Tokyo, Japan). As shown in Figure 2, serum levels of HMGB1 were elevated during acute phase and continued to decrease during subacute phase; these results were consistent with the previous reports (7, 8).

## Discussion

We report the first case of children who developed complete KD after sunburn injury. Released DAMPs, such as HMGB1, due to the severe sunburn injury might be involved in inflammatory vasculitis and the clinical manifestations of KD.

KD is a self-limiting disease, even in patients with KD who have severe CALs (15). During the self-limiting clinical course of KD, the intensity of systemic inflammation in the acute febrile stage of KD gradually increases and reaches a peak (mean 6th day of fever onset) (16). After the acute febrile stage, inflammation gradually decreases and the disease progresses to the convalescent stage (16). Thus, it has been postulated that the host immune reactions preceding this peak of inflammation process mediates tissue cell injury, whereas the immune reactions following the peak mediate tissue cell repair.

The current immunological concept on infectious diseases or infection-associated immune-mediated diseases, including KD, may have limitations to explain the immunopathogenesis of the diseases. However, same host immune cells and immune proteins are involved in all pathologic lesions of the diseases (1, 3). It was once believed that host cell injury in various infectious diseases and immune-mediated diseases is caused solely by pathogens themselves; however, it is now known that host immune reactions and the smaller substances from the infectious agents, including toxins and PAMPs/MAMPs, and/or from the injured host cells by infectious insults such as DAMPs are also involved (1, 3, 17). It is postulated that the main function of the host immune/repair systems is to control the levels of such toxic substances, based on their size and other characteristics. Adaptive immune cells, namely, B cells and T cells, control pathogenic proteins and peptides, respectively, and the innate immune system may control larger complexes such as viruses, bacteria, and apoptotic or necrotic debris and other smaller non-protein substances through phagocytosis and immune proteins, including natural antibodies. Briefly, host immune cells, including macrophage-linage cells, control not only PAMPs/MAMPs but also DAMPs that may be derived from host cells injured by infectious insults (the protein-homeostasis-system hypothesis) (18, 19). This control system of the host may be important for recovery from KD (1, 3, 17). Various pathogen infections and other conditions could be associated with KD inflammation partially via DAMPs (1, 3–8, 17). DAMPs, including HMGB1, could be caused by sun burn injury because excess exposure to ultraviolet radiation in sunlight can result in DNA damage followed by NLRP3 inflammasome activation of the skin (20); this mechanism might be involved in KD inflammation (4–8). However, they could not present direct evidence on the relationship between DAMPs following sun burn injury and KD, which is one of the limitations of our study.

In conclusion, this is a single case, but it is unique since it suggests the inflammatory process of KD, involving the DAMPs.

## Data Availability Statement

The datasets generated for this study are available on request to the corresponding author.

## Ethics Statement

The studies involving human participants were reviewed and approved by Institutional Review Board of Saiseikai Shimonoseki General Hospital. Written informed consent to participate in this study was provided by the participants' legal guardian/next of kin. Written informed consent was obtained from the minor(s)' legal guardian/next of kin for the publication of any potentially identifiable images or data included in this article.

## Author Contributions

SO and SH led conceptualization and design of the analysis, analyzed and interpreted data, and drafted the initial manuscript. AM, YO, RH, SF, and YI contributed to data collection and critically reviewed the manuscript. Each author listed on the manuscript has seen and approved the submission of this version of the manuscript and takes full responsibility for the manuscript. This manuscript has not been published or presented elsewhere in part or in entirety, and is not under consideration by another journal.

### Conflict of Interest

The authors declare that the research was conducted in the absence of any commercial or financial relationships that could be construed as a potential conflict of interest.

## Abbreviations

CALs: coronary artery lesions
CRP: C-reactive protein
DAMPs: damage-associated molecular patterns
HMGB1: high mobility group box 1
IVIG: intravenous immunoglobulin
KD: Kawasaki disease
MAMPs: microbe-associated molecular patterns
PAMPs: pathogen-associated molecular patterns
SD: standard deviation
WBC: white blood cell.
